# Rebuilding the human testis in vitro

**DOI:** 10.1111/andr.12710

**Published:** 2019-09-28

**Authors:** E. Oliver, J.‐B. Stukenborg

**Affiliations:** ^1^ NORDFERTIL Research Lab Stockholm, Childhood Cancer Research Unit Department of Women’s and Children’s Health Karolinska Institutet Karolinska University Hospital Solna Sweden

**Keywords:** fertility preservation, testis, Sertoli cells, Leydig cells, spermatogenesis, spermatogonial stem cells

## Abstract

Increasing rates of male infertility have led to a greater need for relevant model systems to gain further insight into male fertility and its failings. Spermatogenesis and hormone production occur within distinct regions of the testis. Defined by specialized architecture and a diverse population of cell types, it is no surprise that disruption of this highly organized microenvironment can lead to infertility. To date, no robust in vitro system has facilitated full spermatogenesis resulting in the production of fertilization‐competent human spermatozoa. Here, we review a selection of current in vitro systems available for modelling the human testis microenvironment with focus on the progression of spermatogenesis and recapitulation of the testis microenvironment.

## The Need For In Vitro Models

Infertility is estimated to affect approximately 7% of the male population, with the most recent cross‐population study suggesting a prevalence closer to 10% (Krausz, 2011; Datta, *et al.*, 2016). Male infertility can result from defects in both sperm production and sperm delivery associated with a multitude of underlying factors including genetic disease, obstruction of the urogenital tract and trauma (Krausz, 2011). The recently reported decline in sperm counts (more than 50%) over the last 50 years has further raised concern about the effect of modern environmental influences on fertility (Levine *et al.*, 2017). Collectively, these studies highlight the urgent need for relevant model systems to gain further insight into male fertility and its shortcomings.

Cancer treatments are a further recognized disruptor of fertility. Studies have reported a 46% prevalence of infertility in male childhood cancer survivors compared to 18% in siblings (Wasilewski‐Masker *et al.*, 2014; Jahnukainen *et al.*, 2015). Therapeutic treatments can both deplete germ cell numbers directly or indirectly damage the somatic components, which are then unable to support spermatogenesis (Anderson *et al.*, 2015; Stukenborg *et al.*, 2018). Advances in treatment have increased the 5‐year survival rate for childhood cancers to approximately 80% (Ward *et al.*, 2014). With increasing numbers of long‐term survivors, the preservation of fertility and endocrine function has now become an important focus. While sperm cryopreservation is standard practice for adult males, options for those unable to produce spermatozoa (e.g. prepubertal boys) are limited (Picton *et al.*, 2015; Stukenborg *et al.*, 2018). As it stands, the current strategy comprises cryopreservation of testicular tissue or cells containing spermatogonial stem cells (SSCs) for future use in reproductive technologies. Subsequent experimental methodologies can be broadly divided into two approaches: (i) autotransplantation of the tissue or SSCs back to the testes on completion of treatment and (ii) in vitro generation of spermatozoa from tissue fragments or single cells for subsequent use in assisted reproduction (e.g. in vitro fertilization (IVF) or intracytoplasmic sperm injection (ICSI)).

Autotransplantation of testicular tissue is an attractive option and has resulted in the generation of non‐human primate offspring (Fayomi *et al.*, 2019), when translated to the clinic; however, the approach poses the risk of reintroducing tumour cells back to the patient. Xenografting, which comprises the grafting of immature testicular tissue under the back skin of immune‐deficient mice, could overcome this. Despite having resulted in the generation of live offspring (as demonstrated in mice, pigs and monkeys) (Shinohara, *et al.*, 2002; Schlatt, *et al.*, 2003; Kaneko, *et al.*, 2013; Liu, *et al.*, 2016), the risk of infection by murine retroviruses must be considered. An alternative approach comprises the implantation of dissociated testis cells back into the testis. This has led to de novo organization of seminiferous tubules and full spermatogenesis in the monkey (Shetty, *et al.*, 2018). As this technology allows for prior cell sorting, the risk of reintroducing tumour cells could be reduced with particular relevance for prepubertal cancer patients.

While SSC transplantation restores spermatogenesis in non‐human primates (Hermann, *et al.*, 2012), the only reported clinical trial in humans has proven unsuccessful (Radford, *et al.*, 1999; Hermann, *et al.*, 2012). If cancer treatment has led to damage of the somatic environment within the seminiferous tubule; however, SSC or testicular tissue transplantation may not be sufficient to restore fertility. In this case, co‐transplantation of SSCs with functional niche cells, as mentioned above (Shetty, *et al.*, 2018), or factors which have a supporting role may improve the efficiency of fertility restoration. An additional limitation of SSC transplantation comes with the relatively small numbers of SSCs present in the human testis and the limited scope of human SSC propagation in vitro (Sadri‐Ardekani, *et al.*, 2009; Nickkholgh, *et al.*, 2014; Yokonishi & Ogawa, 2016). A further avenue of investigation lies in the in vitro production of gametes from reprogrammed stem cells. While significant progress has been made in the differentiation of human embryonic stem cells (hESCs) and induced pluripotent stem cells (hiPSCs) into the male germline, including haploid, round spermatid‐like cells, further progression through spermatogenesis has yet to be achieved (Eguizabal, *et al.*, 2011; Panula, *et al.*, 2011; Easley, *et al.*, 2012). In vitro production of functional spermatozoa from prepubertal testis could overcome all these limitations. In 2011, Sato and colleagues reported complete in vitro spermatogenesis generating fertilization‐competent spermatozoa using culture of intact immature mouse testicular tissue. To date, however, the generation of functional spermatozoa using this approach has not been replicated in primates.

It is clear novel model systems are required to further elucidate the molecular mechanisms of spermatogenesis, with emphasis on recreation of the somatic microenvironment, to enable propagation and maturation of male germ cells in vitro. These would benefit both investigations into the causes of male infertility as well as the development of future strategies to restore fertility. Here, we will discuss current technologies available for modelling the human testis in vitro with a focus on the progression of spermatogenesis and recapitulation of the testis microenvironment.

## The Testicular Microenvironment In Vivo

**Figure 1:**
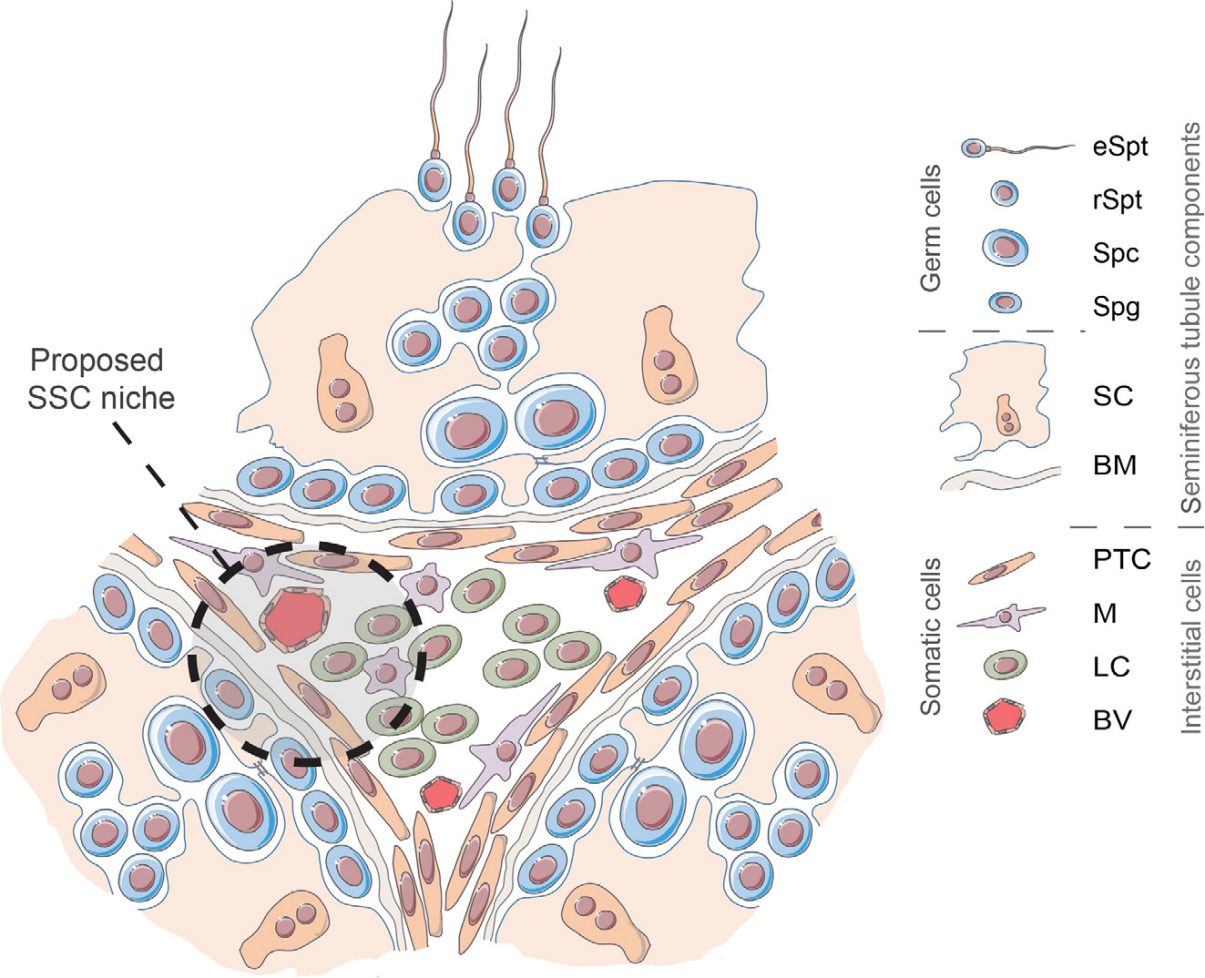
The testicular microenvironment – cell types and organization. The testis is organized into two distinct compartments, the seminiferous tubule and the surrounding interstitial space. The tubule is further separated into basal and luminal compartments by various cellular contacts (e.g. tight, adherence, gap and desmosome) linking together adjacent Sertoli cells (SC) forming the blood–testis barrier. Spermatogenesis takes place in the seminiferous tubule. Spermatogonia (Spg) reside within the basal compartment, while the spermatocytes (Spc), and round (rSpt) and elongating (eSpt) spermatids are contained in the adluminal region. The basement membrane (BM), comprised of extracellular matrix proteins such as fibronectins, laminins and collagens, encloses the seminiferous tubule. Peritubular myoid cells (PTC) line the outside of the basement membrane providing structural support. Interstitial tissue is located between seminiferous tubules and hosts testosterone producing Leydig cells (LC), the vascular network (BV) and various immune cell populations including macrophages (M), of which there are distinct peritubular and interstitial populations. The dashed line represents the proposed spermatogonial stem cell niche highlighting location and various cellular components [Colour figure can be viewed at wileyonlinelibrary.com]

Spermatogenesis is a complex process by which SSCs self‐renew and differentiate into haploid spermatids within the highly specialized architecture of the testis microenvironment. The testes are organized as two structurally discrete compartments, the seminiferous tubule, within which the germ cells differentiate into mature spermatozoa, and the surrounding interstitial space, hosting testosterone producing Leydig cells as well as blood and lymph vessels, nerve fibres, connective tissue and various immune cell populations (Fig. 1) (Oatley & Brinster, 2012). Within the seminiferous tubules, the SSCs reside in the seminiferous epithelium on the basement membrane in distinct locations known as the SSC niche. The niche provides the cues necessary to regulate the fine balance between self‐renewal and differentiation of the SSC population that is required to maintain spermatogenesis throughout the reproductive lifespan (Li & Xie, 2005). The SSC niche is species specific, characterized by distinct organization of the seminiferous epithelium and pattern of spermatogonial development (Fayomi & Orwig, 2018; Hermann, *et al.*, 2018). This is important to consider when selecting an appropriate system in which to model male germ cell differentiation and testicular function in vitro.

The somatic Sertoli cells are generally regarded a fundamental component of the niche given both their location within the seminiferous tubule and direct association with the SSCs. Anchored to the basement membrane, they are in intimate association with germ cells at various stages of maturation. Here, they secrete important growth factors critical for the proliferation and renewal of SSCs including glial cell line‐derived neurotropic factor (GDNF), in addition to fibroblast growth factor 2 (FGF2) and colony‐stimulating factor 1 (CSF‐1) (De Rooij, 2009). GDNF is the primary factor regulating SSC self‐renewal both in vivo and in vitro. In the testis of mice overexpressing GDNF, increased numbers of undifferentiated spermatogonia are observed, whereas germ cell numbers become depleted in GDNF‐deficient mice, indicating an inability of undifferentiated spermatogonia to sustain the germline (Meng *et al.*, 2000). FGF2 has been shown to influence SSC renewal in a similar manner with evidence to suggest a combination of both GDNF and FGF2 is required for SSC renewal in vivo (Takashima, *et al.*, 2015). Sertoli cells further produce factors necessary for differentiation such as activin A, bone morphogenetic 4 (BMP4) and stem cell factor (SCF) (De Rooij, 2009).

Sertoli cells are a tall columnar cell type which extend from the basement membrane to the lumen of the seminiferous tubule (Mruk & Cheng, 2000). Composed of a basally located nucleus and cytoplasmic branches, which extend laterally from a main cytoplasmic stalk, the Sertoli cells surround and support the developing germ cells. The blood–testis barrier is located at the basal region of the Sertoli cells and compartmentalizes the seminiferous tubule into the basal compartment and the adluminal region. Built upon various cellular contacts (e.g. tight, adherence, gap and desmosome), the junctional barrier confers both morphological and functional cell polarization, creating distinct microenvironments that regulate SSC self‐renewal or differentiation through polarized secretion of Sertoli cell proteins (Mruk & Cheng, 2000). Outside the seminiferous tubule, the interstitial tissue provides further key components of the stem cell niche, contributing various growth factors and signalling molecules. Histological examination of testis cross sections from adult mice and rats by Chiarini‐Garcia and colleagues revealed a higher concentration of undifferentiated spermatogonia in areas of the seminiferous tubules which closely associate with the interstitial tissue as oppose to regions of intertubular contact (Chiarini‐Garcia *et al.*, 2001, 2003). The composition of the interstitial region is diverse. The basement membrane, which is in direct contact with both the spermatogonia and Sertoli cells, encloses the seminiferous tubules. Composed of extracellular matrix (ECM) proteins such as fibronectins, laminins and collagens, the basement membrane provides both structural support and mediates local cell signalling effects through binding and release of growth factors to regulate the niche (Hadley & Dym, 1987; Hynes, 2009). Peritubular myoid cells line the outside of the seminiferous basement membrane providing structural support and peristaltic action critical for transport of immotile spermatozoa. In humans, the peritubular cells also possess secretory roles producing GDNF as well as ECM components biglycan and decorin which serve both structural and immunomodulatory functions (Mayerhofer *et al.*, 2018). The closely associated vascular endothelium is thought to play a role in both establishing and maintaining the SSC niche. Using a mouse model, Yoshida et al. describe a preferential localization of undifferentiated spermatogonia to regions of the seminiferous tubule that border vascularized areas of the interstitium (Fig. 1) (Yoshida *et al.*, 2007). More recent investigation suggests that testicular endothelial cells provide the necessary growth factors, including GDNF, for the self‐renewal and expansion of human and mouse SSCs in culture (Bhang *et al.*, 2018).

The steroidogenic Leydig cells populate the interstitium where they produce testosterone which regulates spermatogenesis via action on androgen receptor expressing cells (e.g. peritubular cells and Sertoli cells). Additionally, CSF‐1, produced by Leydig cells, has been demonstrated to promote self‐renewal of SSCs which is perhaps unsurprising given their proximity to the SSC niche (Oatley *et al.*, 2009). Macrophages are a further cell population abundant in the testicular interstitium often associated with the vascular endothelium (Hume *et al.*, 1984). In addition to their immune roles, interstitial macrophages form close contacts with Leydig cells secreting intermediate compounds necessary for steroid biosynthesis (Hutson, 2006). A further distinct population of macrophages localize along the seminiferous tubules in regions enriched for undifferentiated spermatogonia where they regulate spermatogonial differentiation through expression of growth factors such as CSF‐1 (Defalco *et al.*, 2015).

A functioning testicular microenvironment is critical for normal spermatogenesis and reproductive function. As previously discussed, damage to the testicular microenvironment can lead to impaired fertility due to a loss of germ cell support (Anderson *et al.*, 2015; Stukenborg *et al.*, 2018). Further understanding of the unique characteristics of the human testicular microenvironment (organization, cell types, integration of signalling cues) will therefore be critical to our finally achieving full human spermatogenesis in vitro.

## 2D In Vitro Models

**Figure 2:**
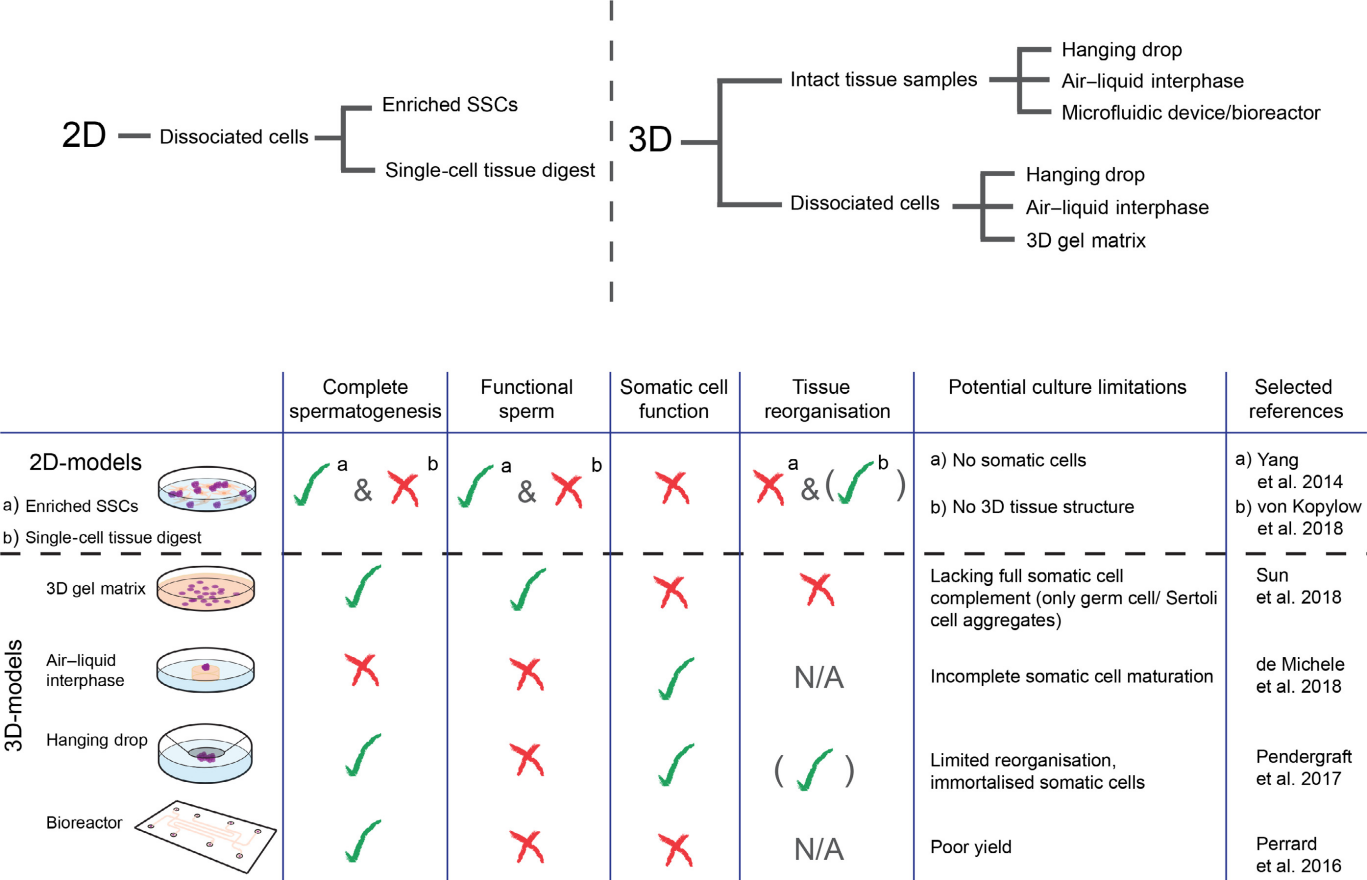
Schematic overview highlighting functionality and limitations of select in vitro human testis models. For the purpose of the review, we have defined complete spermatogenesis as the production of elongating spermatids from spermatogonial stem cells. Sperm functionality is based on proven ROSI fertilization of a mouse oocyte. Hormone production in culture (e.g. testosterone, anti‐Müllerian hormone) indicates somatic cell function. Tissue reorganization is based on the formation of seminiferous tubule‐like structures or the blood–testis barrier similar to in vivo organization. Demonstrated functionality (green tick), partial functionality (green tick in brackets), not demonstrated (red cross) [Colour figure can be viewed at wileyonlinelibrary.com]

Numerous 2D culture models have been employed to explore the conditions required for complete spermatogenesis (Fig. 2). A popular approach has been the culture of SSCs; however, such attempts are limited by an inability to isolate pure populations of SSCs due to a lack of defined markers. While a recent spate of single‐cell transcriptome studies has led to the identification of four undifferentiated human spermatogonial cell clusters, allowing for purification of the most primitive (and likely SSC‐enriched) cell subset, a unique SSC gene expression signature still remains elusive (Li *et al.*, 2017; Guo *et al.*, 2018; Hermann *et al.*, 2018; Wang *et al.*, 2018; Sohni *et al.*, 2019). Studies are further limited by the low number of SSCs in the testis, which range from 0.01% to 1% of all testicular cells (Tegelenbosch & De Rooij, 1993). While stable proliferation over several months has been achieved in various mammalian species (Kanatsu‐Shinohara *et al.*, 2003; Hamra *et al.*, 2005), a long‐term expansion system has yet to be established in humans, with culture and propagation in vitro currently reported up to 28 weeks (Sadri‐Ardekani *et al.*, 2009). Nevertheless, Yang et al. report retinoic acid (RA) and SCF stimulated generation of haploid spermatids from isolated human SSCs in conventional single cell culture (Yang *et al.*, 2014).

Progression of spermatogenesis from post‐spermatogonial developmental stages has additionally been described. Co‐culture with Vero cells or Sertoli cells as a cell feeder layer allows for generation of elongating fertilization‐competent spermatids from round spermatids with increased efficacy observed with follicle stimulating hormone (FSH) supplementation (Tesarik *et al.*, 1998; Tesarik *et al.*, 1998; Cremades *et al.*, 1999; Sousa *et al.*, 2002). These findings were subsequently extended using only conditioned medium from Vero cells (Cremades *et al.*, 2001), while Tanaka et al. later demonstrated completion of meiosis describing the generation of round spermatids from primary spermatocytes in culture with Vero cells (Tanaka, *et al.*, 2003). Further to supporting germ cell development, somatic cell interactions are necessary for establishing testicular structural organization in culture. Two‐dimensional co‐culture of rodent Sertoli cells with peritubular myoid cells promotes Sertoli cell reorganization, generating seminiferous tubule‐like structures in vitro (Tung & Fritz, 1980), while Sertoli cells from rats grown on ECM deposited by Sertoli‐myoid cell co‐cultures establish polarized monolayers on which germ cells can be maintained for up to 5 weeks in contrast to the squamous monolayer formed when cultured on plastic alone (Hadley *et al.*, 1985). Tubular reorganization in these cultures is driven by fibronectin, a component of the basement membrane synthesized by myoid cells, which promotes the migration of Sertoli cells (Tung & Fritz, 1986; Richardson *et al.*, 1995). Despite achieving basic structural reorganization, these studies result in limited germ cell development, suggesting a full complement of somatic testicular cell types is required for the progression of spermatogenesis in culture. A more recent approach describes the culture of single cell digested adult testicular biopsies on uncoated culture dishes in a 2D system (Mincheva *et al.*, 2018; Von Kopylow *et al.*, 2018). While permitting cultivation of various somatic testicular cells over 12 weeks, the reorganization of seminiferous cord‐like structures was limited and insufficient to support the maintenance of germ cells which were often located outside the cell clusters (Von Kopylow *et al.*, 2018). Dedifferentiation of somatic cell types was speculated to underlie the failure of germ cells to incorporate into the cellular aggregates (Von Kopylow *et al.*, 2018).

Two‐dimensional in vitro culture systems therefore fail to replicate the complete process of spermatogenesis, providing an insufficient niche for long‐term germ cell maintenance, propagation and differentiation. Despite this, many of the culture conditions employed are necessary to recapitulate aspects of the testis microenvironment and are common to a number of the models covered in this review (Table 1). In most mammals, including humans, spermatogenesis requires a temperature 2–3°C below core body temperature (Newman & Wilhelm, 1950). Given this, cultures are routinely maintained at 33–35°C, with greater temperatures having a deleterious effect on the viability of spermatogonia and Sertoli cells (Medrano, *et al.*, 2018). Supplementation of culture medium with pituitary gonadotrophins luteinizing hormone (LH) or FSH promotes Sertoli cell survival and maturation of spermatogonia up to meiotic initiation (Tesarik *et al.*, 1998; Medrano *et al.*, 2018), while various other factors are necessary to maintain the balance between SSC renewal (GDNF) and differentiation (RA) (De Rooij, 2009; Yang, *et al.*, 2014).

**Table 1:** Experimental details of select in vitro human testis studies and resulting progression of spermatogenesis

Culture model	Age of tissue donor	Progression of spermatogenesis	Medium	Serum	Additional factors	Gonadotrophins	Scaffold	Temperature/CO_2_	Selected references
2D – Enriched SSCs	Adult	SSC – rSpt	DMEM/F12	10% FBS	RA (2 µm) SCF (20–150 ng/ml)	–	–	34°C 5%	Yang *et al. *(2014)
2D – Single‐cell tissue digest	Adult	Presence of eSpt	DMEM	15% KSR	2‐Mercaptoethanol (0.1 mm) L‐Glutamine (2 mm) 100 × MEM non‐essential amino acids (0.1 mm) EGF (40 ng/ml) FGFa (20 ng/ml) FGFb (20 ng/ml) FGF9 (20 ng/ml) GDNF (100 ng/ml) IGF (10 ng/ml)	–	–	35°C 5%	Von Kopylow *et al. *(2018)
3D – Gel matrix	Peripubertal – adult	SSC – rSpt	DMEM/F12	10% KSR	RA (2 µm) SCF (100 ng/ml) BMP4 100 ng/ml) Testosterone (10^−6^ m)	–	Matrigel	34°C 5%	Sun *et al. *(2018)
3D – Air–liquid interphase	Prepubertal	Diploid to haploid germ cells	DMEM/F12	10% KSR	L‐Glutamine (0.35 mg/mL) Retinol (10^−6^ m) Vitamin C (0.05 mg/ml) Pyruvate (0.0025 m) Triiodothyronine (T3) (5 pmol/L) GDNF (10 ng/ml) 22(R)‐hydroxycholesterol (20 mm/L) Prolactin (5 ng/ml)	FSH (5 UI/L) hCG (1 UI/L)		34°C 5%	de Michele *et al. *(2018)
3D – Hanging drop	Adult	Diploid to haploid germ cells	StemPro‐34	–	RA (2 µm) SCF (100 ng/ml)	FSH (2.5 × 10^−5^ IU)	Hanging drop + ECM extract (1 µg/ml)	34°C 5%	Pendergraft *et al. *(2017)
3D – Bioreactor	Adult	Generation of morphologically mature spermatozoa	DMEM/F12	–	NaHCO_3_ (1.2 g/L) Insulin (10 µg/ml) Transferrin (10 µg/ml) Vitamin C (10^−4^ m) Vitamin E (10 µg/ml) RA (3.3 × 10^−7^) Retinol (3.3 × 10^−7^) Pyruvate (10^−3^) Testosterone (10^−7^)	FSH (1 ng/ml)	Hydrogel	33°C 5%	Perrard *et al.* (2018)

BMP, bone morphogenetic protein; DMEM, Dulbecco’s modified Eagle’s medium; EGF, epidermal growth factor; eSpt, elongating spermatid; FBS, fetal bovine serum; FGF, fibroblast growth factor; GDNF, glial cell line‐derived neurotrophic factor; IGF, insulin‐like growth factor; KSR, KnockOut serum replacement; RA, retinoic acid; rSpt, round spermatid; SCF, stem cell factor; SSC, spermatogonial stem cell.

## 3D In Vitro Models

Culture of intact testes fragments exploits the native organization of the tissue conserving structural support and cellular interactions which cannot be achieved in a 2D system (Fig. 2). The pioneering studies of testes tissue culture were performed by Steinberger who described the culture of rat (Steinberger, *et al.*, 1964; Steinberger & Steinberger, 1965), and later, human testicular tissue (Steinberger, 1967) using an air–liquid interface method. Adapted from Trowell, the system comprises the suspension of tissue fragments that are partially immersed in culture medium on a semi‐solid support, balancing the delivery of nutrients from the culture medium with efficient gas exchange (Trowell, 1954). Applying the same principle, Sato and colleagues achieved complete spermatogenesis in vitro nearly 50 years later, generating fertilization‐competent spermatozoa from immature mouse testis fragments (Sato *et al.*, 2011). While the success of the system is likely underpinned by maintaining the native testis microenvironment, the complete maturation of spermatozoa appears dependent on the medium component knockout serum replacement (KSR). Using the same system, subsequent studies from our group revealed that a minimum concentration of 10% KSR is required to induce both full spermatogenesis and testosterone production (Reda *et al.*, 2017). The latter of which may be explained by the lipid‐rich cholesterol component of KSR which is the precursor for testosterone synthesis (Garcia‐Gonzalo & Izpisua Belmonte, 2008). While the exact factors required for germ cell differentiation remain undefined, studies suggest testosterone is necessary to sustain both the meiotic process and germ cell survival (especially in the later stages of differentiation) (Erkkila *et al.*, 1997; Tesarik *et al.*, 1998). Sato and colleagues, however, further noted maturation of the somatic Sertoli cells and peritubular myoid cells within the culture system, as illustrated by the onset of androgen receptor expression, demonstrating their maturity to support spermatogenesis (Sato *et al.*, 2011). This suggests that the system may in fact be enabling spontaneous spermatogenesis by providing germ cells with an appropriate niche instead of simply supplying the growth factors necessary for differentiation.

Despite the subsequent success in other species, for example rats (Reda *et al.*, 2016), the air–liquid interface system has yet to been refined for human application, although partial spermatogenesis has been described. Roulet et al. report differentiation of primary spermatocytes from the preleptotene to pachytene stage in cultured adult testis obtained from prostate cancer patients, however, observed a progressive loss of meiotic and post‐meiotic cells within the 16‐day culture (Roulet *et al.*, 2006). Using the same system, de Michele and colleagues describe preserved seminiferous tubule structure and somatic cell function capable of maintaining spermatogonial proliferation for up to 139 days in prepubertal testis tissue (De Michele *et al.*, 2017). Leydig cell functionality was demonstrated by testosterone production which peaked at 10 days in culture while the observed decrease in anti‐Müllerian hormone expression suggests Sertoli cell maturation. While the approach preserves paracrine interactions facilitating maturation of the somatic cell niche, germ cell differentiation was not achieved. These findings were recently extended with reports of haploid cells generated from immature testis tissue (De Michele *et al.*, 2018). Inclusion of factors required for both SSC renewal (e.g. GDNF) and differentiation (e.g. retinoic acid) in the differentiation medium allowed for accelerated maturation of the SSC niche supporting germ cell differentiation. Whether these cell types are capable of differentiating further into fertilization‐competent spermatozoa is yet to be defined.

Three‐dimensional culture systems are an alternative strategy used to re‐engineer the in vivo testis microenvironment. Such approaches are broadly classified as scaffold‐free or scaffold‐based and can comprise natural or synthetic materials. Scaffold‐free systems primarily consist of the formation of multi‐cellular aggregates, an example of which is the hanging drop system. Based on studies described in 1907 by Harrison, the method has often been used for the generation of embryoid bodies from human embryonic stem cells (Harrison *et al.*, 1907; Brickman & Serup, 2017). Comprising the suspension of cells in a small volume of medium on the underside of the culture dish lid, the system facilitates the formation of 3D aggregates at the apex of the droplet of medium. Using this approach in combination with solubilized human testis, ECM has resulted in generation of a functional testicular system by co‐culture of adult human SSCs and immortalized human Leydig and Sertoli cells (Pendergraft *et al.*, 2017). While reorganization was limited, germ cells were associated within the centre of resulting clusters with somatic cells located in the periphery. The authors report organoid functionality for three weeks demonstrating both testosterone production and generation of haploid germ cells albeit at a low frequency (0.2%). The system has since provided useful insight into the mechanism of Zika infection in the testes, demonstrating decreased organoid viability and reduced expression of spermatogonial and somatic cell markers upon infection (Siemann *et al.*, 2017; Strange *et al.*, 2018). More recently, Sakib and colleagues reported a microwell aggregation approach, generating organoids from mouse (postnatal day 8–10), pig (1 week) and human prepubertal tissue (5 months and 5 years) (Sakib *et al.*, 2019). While comprising various testicular cell types (germ cells, Sertoli cells, Leydig cells and peritubular myoid cells) and defined interstitial and seminiferous epithelium compartments, the structures assumed a reverse polarity whereby the seminiferous epithelium was situated at the surface with the interstitial compartment located within the organoid (Sakib *et al.*, 2019). Despite this, a high level of structural organization was observed when compared to previously characterized human organoids; however, the use of tissue from older donors (adult) in earlier studies (Pendergraft *et al.*, 2017) likely underlies the more limited reorganization.

In an alternative approach, Jorgensen et al cultured intact testicular fragments within a hanging drop system, reporting maintenance of three‐dimensional tissue architecture and survival and proliferation of germ cells for up to 14 days (Jorgensen, *et al.*, 2014). Despite the limited culture duration, the approach requires minimal media and supplements offering an efficient system to investigate the development of human germ cells retained with the testicular microenvironment (Jorgensen *et al.*, 2014; Jorgensen *et al.*, 2015; Jorgensen *et al.*, 2018). The most recent study by Jorgensen and colleagues employed the hanging drop method in combination with xenografting to study the role of the Nodal/Activin pathway in the first‐ and second‐trimester human foetal gonad development. Here, they revealed inhibition of Nodal signalling resulted in decreased gonocyte numbers and impaired seminiferous cord formation, the latter of which could be rescued over time following testicular tissue grafting (Jorgensen *et al.*, 2018).

Precise topological organization of the testis is crucial for compartmentalizing signalling cues which drive cell fate decisions (Lord *et al.*, 2018). While supporting partial human spermatogenesis, the described 3D systems fail to fully replicate the specialized architecture of the testicular microenvironment. Scaffold‐based culture strategies offer an alternative approach. One such example is the use of a biocompatible decellularized testicular matrix. In an effort to induce de novo formation of testicular architecture, Baert and colleagues combined isolated cell suspensions from either adult or prepubertal donors with a decellularized human cadaveric testicular matrix (Baert *et al.*, 2015, 2017). While retaining native 3D tissue structure and testis‐specific ECM components, such as collagen, laminin and fibronectin, the scaffold was unable to offer a more favourable environment for tissue reorganization (Baert *et al.*, 2015, 2017). Despite this, the primary testicular cells from adult and pubertal tissue demonstrated self‐assembly into spheroid structures. While lacking testis‐specific topography, the resulting organoids supported the maintenance and propagation of germ cells throughout culture and retained somatic cell functionality, as demonstrated by testosterone and inhibin B production (Baert *et al.*, 2017). Additional scaffold approaches have been used to explore the de novo arrangement of testis structure. Collagen sponges, which offer a porous 3D structure with an ECM like surface, allow for partial reorganization of tubule‐like structures following colonization by dissociated rat testicular cells (Reuter *et al.*, 2014). While topological cues in the form of silicon‐based nanostructures can direct cord‐like formation by aligning peritubular cell and Sertoli cell bodies in the direction of nanogratings (Pan *et al.*, 2013). Synthetic scaffolds such a silicon also offer a defined chemical composition allowing for reproducibility however lack sites for cellular adhesion and often require a coating of ECM proteins in an attempt to mimic the nice in which cells reside naturally (Knight & Przyborski, 2015). Despite the reported lack of germ cell differentiation, such approaches are relevant for better understanding the mechanisms of testicular cord formation.

Although 3D culture systems favour progression of spermatogenesis and testicular reorganization, it appears combination with ECM components provides a more desirable environment. Encapsulation of dissociated testicular cells from azoospermic adult patients in collagen gel matrix results in cellular aggregation sufficient for the differentiation of spermatids from spermatocytes (Lee *et al.*, 2007). The same system potentiates the meiotic progression and post‐meiotic differentiation of rodent germ cells (Lee *et al.*, 2006). In a recent study, Sun and colleagues report the generation of post‐meiotic cells from SSCs isolated from azoospermic patients using a 3D approach (Sun *et al.*, 2018). Comprised of purified SSCs co‐cultured with Sertoli cells in a Matrigel matrix, the system supports germ cell differentiation producing functional haploid spermatids with the capacity to fertilize mouse oocytes. Inclusion of factors essential for SSC renewal and differentiation including RA, SCF, BMP4 and testosterone, in addition to KSR (10%), allowed for increased differentiation efficiency, 10‐fold greater than previously reported by Sato et al (Sato *et al.*, 2011). Studies therefore suggest the effect of ECM is further pronounced when cells are embedded within a gel matrix, such as collagen or Matrigel (Sun *et al.*, 2018), as oppose to simply occupying cavities of a scaffold (Pan *et al.*, 2013; Reuter *et al.*, 2014; Baert *et al.*, 2017). In addition to ECM however, other matrices (e.g. agarose, methylcellulose) have been used with similar success, highlighting the principle need of an encapsulating 3D structure to initiate male germ cell maturation in vitro (Stukenborg *et al.*, 2008, 2009; Abu Elhija *et al.*, 2012).

In a novel approach to 3D culture, we have recently detailed a multilayer model termed the three‐layer gradient system (3LGS) to generate rat testicular organoids in vitro (Alves‐Lopes et al., 2017, 2018). Using a multilayer approach, whereby testicular cell suspensions are embedded in a layer of Matrigel situated between two cell‐free layers, we report the reorganization of seminiferous tubule‐like structures with a functional blood–testis barrier supporting germ cell maintenance and proliferation up to 21 days in culture. Uniquely, the system generates a whole organ structure, comprising multiple tubule‐like structures situated within an interstitial environment as oppose to individual clusters as described in other studies (Baert *et al.*, 2017; Pendergraft *et al.*, 2017; Sakib *et al.*, 2019). We propose that the success of system centres on the generation of two concentration gradients formed by the layered structure – the inflow of factors from the Matrigel (to be consumed by the cells) and the subsequent outflow of cellular metabolites. Supporting this hypothesis, tubule‐like structures do not reorganize in a single layer of Matrigel using the same volume and cell concentration as used in the 3LGS (Alves‐Lopes *et al.*, 2017).

Alternative dynamic culture approaches have been described in an attempt to promote germ cell differentiation in vitro. Using a bioreactor system, Perrard and colleagues report differentiation of morphologically mature spermatozoa from spermatogonia in human seminiferous tubule fragments enclosed in porous chitosan hydrogel tubes immersed in culture medium (Perrard *et al.*, 2016). Whether the resulting cell types are fertilization‐competent has yet to be defined. Building on the success of the air–liquid interface method (Sato *et al.*, 2011), Komeya et al describe the development of a microfluidic device (Komeya *et al.*, 2016). Composed of a continuously flowing medium channel separated from a tissue chamber by a nutrient permeable membrane, the authors report maintenance of murine testis fragments and complete spermatogenesis for up to 6 months. The success of the described systems over the previous static organ‐culture approach is likely underpinned by the dynamic exchange of nutrients, waste and gas. Normally regulated by the microvasculature, both systems allow for locally established homoeostasis through molecular diffusion similar to the blood plasma‐interstitial tissue relationship in vivo. Dynamic culture may also confer a gradient of paracrine and autocrine factors formed in the basal, adluminal and luminal compartments of the seminiferous tubes. While we recapitulated this to some extent with our 3LGS, the approach is limited in regard to tissue size (resulting in the formation of central necrotic areas) and duration of culture, perhaps due to the saturation of supporting Matrigel. Lack of vascularization is a limitation of organoid technologies, particularly when considering the generation of tissue for clinical transplantation. Given the success in other fields, one approach may be the supplementation of cultures with an exogenous source of endothelial and mesenchymal progenitor cells in order to induce tissue vascularization (Takebe *et al.*, 2015). Further studies will be required to fully explore the 3LGS system as a model for the testis.

## Conclusions

A functioning somatic microenvironment is crucial for the process of spermatogenesis. When considering in vitro modelling of the human testis with the intent of generating mature spermatozoa, this must be a primary consideration. Studies that have made the most progress have either exploited the intrinsic microenvironment using testis tissue fragments or encapsulated dissociated cells within a supportive matrix to generate a 3D structure. In the effort to replicate human spermatogenesis in vitro, future focus should be on maintaining the functional niche or, in the case of immature tissue, maturation of the niche to support spontaneous spermatogenesis.
